# Coronary artery aneurysm, ectasia and stenosis in a 53-year-old man with HIV infection

**DOI:** 10.1093/jscr/rjac056

**Published:** 2022-03-04

**Authors:** Abhishek Kashyap, Dmitry Abramov, Aditya Bharadwaj, Miriam Rabkin, David G Rabkin

**Affiliations:** Department of Cardiothoracic Surgery, Loma Linda University Medical School, Loma Linda, CA, USA; Department of Medicine (Division of Cardiology) Loma Linda University Medical School, Loma Linda, CA, USA; Department of Medicine (Division of Cardiology) Loma Linda University Medical School, Loma Linda, CA, USA; Mailman School of Public Health, Columbia University Medical Center, New York, NY, USA; Department of Cardiothoracic Surgery, Loma Linda University Medical School, Loma Linda, CA, USA

**Keywords:** human immunodeficiency virus, coronary artery disease, coronary artery aneurysm, coronary artery bypass graft

## Abstract

The impact of long-standing human immunodeficiency virus infection (HIV) and potent anti-retroviral therapy on the coronary circulation is unknown; however, scattered reports are emerging of coronary aneurysms in this population. We report what we believe to be the first described case of both coronary stenosis and coronary artery aneurysms in a person living with HIV and discuss management options.

## INTRODUCTION

The availability of potent antiretroviral therapy (ART) has markedly extended the life expectancy of people living with HIV (PLHIV) [1, 2], and non-communicable diseases including cardiovascular disease are increasingly common causes of morbidity and mortality for people with access to ART [3, 4]. PLHIV are at higher risk of coronary artery disease (CAD) than their HIV-negative peers [5, 6], likely due to the direct effects of HIV on inflammatory and coagulation markers [7, 8] and the effect of some antiretroviral medications on CAD risk factors including lipodystrophy, dyslipidemia and insulin resistance [9]. Infection by HIV also results in diffuse endothelial dysfunction affecting multiple organ systems including the coronary arteries [10]. Although less is known about the association between HIV and coronary aneurysms, extra-cranial arterial aneurysms in the context of HIV have been well-described [11], and coronary artery aneurysms have been reported in patients with acquired immunodeficiency syndrome [12, 13] and other vasculitides [14, 15]. We report what we believe to be the first described case of both coronary stenosis and coronary artery aneurysms in a person living with HIV.

## CASE REPORT

A 53-year-old HIV+ man on ART since 2005 with a CD4 count of 700 cells/μl, an undetectable viral load and a past medical history significant for hypertension, dyslipidemia, obesity and gout presented to an outside hospital after developing left arm numbness and tingling which woke him from sleep. In addition to his ART, he was taking a beta blocker and a statin. He did not smoke tobacco or use illicit drugs and had no family history of CAD or connective tissue disorder. He had no history of dysphagia or stridor.

**Figure 1:**
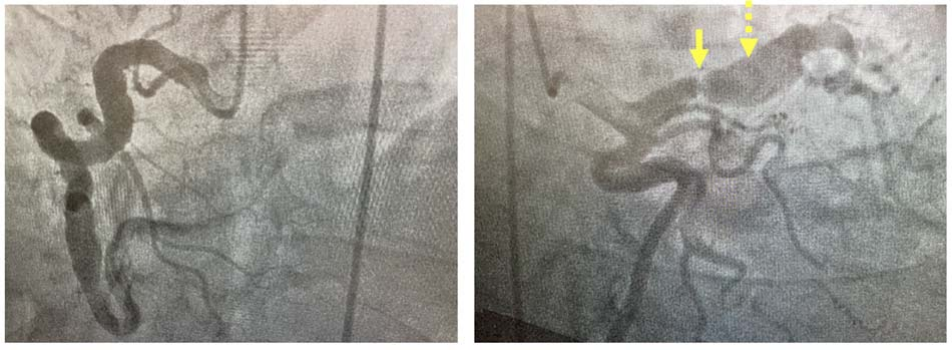
Coronary angiogram. Left panel: injection of fusiform aneurysm of right coronary artery. Right panel: coronary angiogram injection of the left-sided coronary circulation with solid yellow arrow pointing to tight left anterior descending stenosis and dotted yellow arrow pointing to post-stenotic aneurysm.

**Figure 2:**
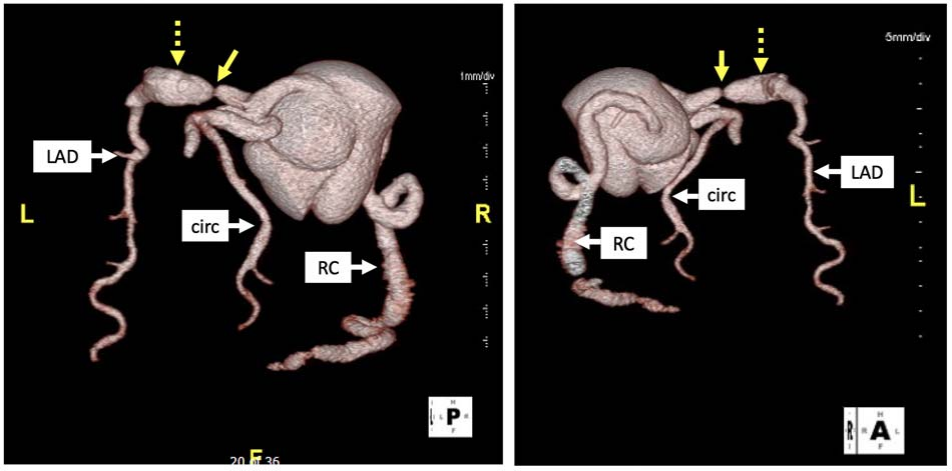
Computed tomography with 3D reconstruction. Solid yellow arrow points to tight stenosis in the left anterior descending and dotted yellow arrow points to post-stenotic aneurysm.

His initial troponin was not elevated although the electrocardiogram showed new ST depressions in the medial precordial leads and subsequent troponins were elevated. He was transferred to our institution and underwent left heart catheterization which demonstrated a right dominant circulation with ectasia of the right coronary artery, a fusiform aneurysm of the left anterior descending coronary artery (LAD) and a generous-sized left circumflex coronary artery (Fig. 1). There was a 99% stenosis of the LAD with post-stenotic dilatation up to 13 mm with gradual normalization over the course of the remainder of the vessel. An echocardiogram showed a left ventricular ejection fraction of 35% without valvular pathology. Percutaneous coronary intervention (PCI) was thought to be a poor option because the distal portion of the stent would not oppose the wall of the vessel but be ‘floating’ in the aneurysmal portion of the artery pre-disposing thrombus formation. Therefore, in anticipation of operative intervention, a computed tomography scan was done to further define the anatomy. The scan demonstrated extensive wall thickening and contour irregularity suggestive of diffuse coronary arteritis (Fig. 2). The study also showed a dilated main pulmonary artery up to 46 mm and an aberrant right subclavian artery coming off the distal aortic arch and passing behind the esophagus.

**Figure 3:**
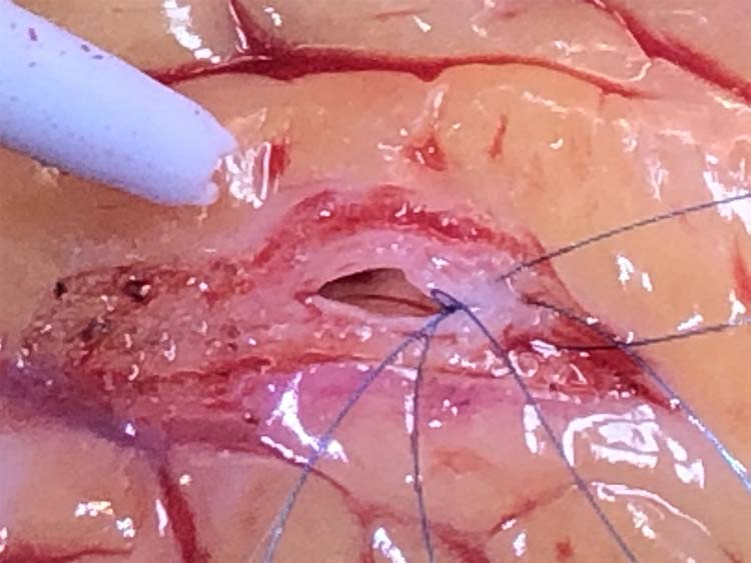
Intra-operative appearance of thickened and rubbery LAD.

He was then brought to the operating room for coronary artery bypass grafting (CABG) and underwent an uneventful left internal mammary artery bypass to LAD. The anastomosis was in the relatively normal-sized mid-portion of the LAD which was found to be rubbery in consistency and thick-walled (Fig. 3). His ventricular function normalized on intra-operative trans-esophageal echocardiogram. The patient had an uneventful recovery and, after several days, was discharged on Plavix and aspirin in addition to his pre-operative medications which included a beta blocker and a statin. He was doing well at last follow-up.

## DISCUSSION

Coronary artery aneurysms are found in about 5% of patients presenting for coronary angiography, while multiple aneurysms are rarer [16]. Atherosclerosis accounts for over 90% of coronary artery aneurysms in adults; other etiologies include infectious causes, connective tissue disorders, cocaine use, trauma and inflammatory disorders such as Kawasaki’s disease, Takayasus arteritis, Giant cell arteritis and Behcet’s disease. Coronary angiography is the mainstay of diagnosis. Some confusion exists regarding terminology; aneurysm generally refers to a focal dilatation greater than 50% of the normal diameter of the vessel; a true aneurysm involves all three vascular layers, while a false aneurysm is composed only of the adventitia. Saccular aneurysm have a transverse diameter greater than longitudinal diameter, while fusiform aneurysms are the opposite. Coronary ectasia is a diffuse dilatation of the vessel. Complications of coronary artery aneurysms include thrombus formation, fistula formation, dissection and, less commonly, rupture.

Algorithms for the management of coronary artery aneurysms are not well-defined. Treatment options include surgical, percutaneous and medical therapies. For patients with atherosclerotic coronary disease and coronary aneurysms, CABG is a well-defined management option based on the Coronary Artery Surgery Study registry (>500 patients), and no difference was found in outcome among patients undergoing CABG with and without coronary aneurysms [16]. Other surgical options include aneurysm ligation, resection or marsupialization with an interposition graft. Percutaneous therapy includes stenting and/or coiling. A comparison of surgery and stenting of coronary aneurysms found no deaths in either group but higher rate of incidence of reintervention in the stenting group [17]. Medical management includes blood pressure control and anti-platelet therapy.

Scattered case reports have begun to emerge in the literature of PLHIV with coronary artery aneurysms [12, 13]. Putative explanations include autoimmune inflammation causing intimal disease or disruption and ART-related atherosclerotic disease leading to aneurysm formation. Since initial reports dispelled concerns about the immunosuppressive effect of cardiopulmonary bypass in the context of HIV infection [18], CABG has emerged as routine therapy in the HIV+ population. Short-term outcomes are identical to matched HIV− controls; however, long-term repeat revascularization is more common in HIV+ patients due to *de novo* disease in the native coronary arteries [19]. Because the patient’s anatomy was felt to be suboptimal for PCI, we felt comfortable with surgical revascularization. We elected not to exclude or marsupialize the coronaries because doing so would have required reconstruction of almost all the patient’s entire epicardial coronary circulation with unproven benefit. We did not translocate the aberrant subclavian artery because it was asymptomatic.

## CONFLICT OF INTEREST STATEMENT

None declared.

## FUNDING

None.

## References

[1] Samji H, Cescon A, Hogg RS, Modur SP, Althoff KN, Buchacz K, et al. Closing the gap: increases in life expectancy among treated HIV-positive individuals in the United States and Canada. PLoS One 2013;8:e81355. 24367482 10.1371/journal.pone.0081355 PMC3867319

[2] Teeraananchai S, Kerr SJ, Amin J, Ruxrungtham K, Law MG. Life expectancy of HIV-positive people after starting combination antiretroviral therapy: a meta-analysis. HIV Med 2017;18:256–66. 27578404 10.1111/hiv.12421

[3] de Coninck Z, Hussain-Alkhateeb L, Bratt G, Ekström AM, Gisslén M, Petzold M, et al. Non-AIDS mortality is higher among successfully treated people living with HIV compared with matched HIV-negative control persons: a 15-year follow-up cohort study in Sweden. AIDS Patient Care STDS 2018;32:297–305. 30067408 10.1089/apc.2018.0015 PMC6088250

[4] Harris TG, Rabkin M, El-Sadr WM. Achieving the fourth 90: healthy aging for people living with HIV. AIDS 2018;32:1563–9. 29762172 10.1097/QAD.0000000000001870 PMC6082594

[5] Triant VA, Lee H, Hadigan C, Grinspoon SK. Increased acute myocardial infarction rates and cardiovascular risk factors among patients with human immunodeficiency virus disease. J Clin Endocrinol Metab 2007;92:2506–12. 17456578 10.1210/jc.2006-2190 PMC2763385

[6] Paisible AL, Chang CC, So-Armah KA, Butt AA, Leaf DA, Budoff M, et al. HIV infection, cardiovascular disease risk factor profile and risk for acute myocardial infarction. J Acquir Immune Defic Syndr 2015;68:209–16. 25588033 10.1097/QAI.0000000000000419 PMC4441201

[7] Kaplan-Lewis E, Aberg JA, Lee M. Atherosclerotic cardiovascular disease and anti-retroviral therapy. Curr HIV/AIDS Rep 2016;13:297–308. 27562769 10.1007/s11904-016-0331-y

[8] Aberg JA . Aging, inflammation, and HIV infection. Top Antivir Med 2012;20:101–5. 22954610 PMC6148943

[9] Kiage JN, Heimburger DC, Nyirenda CK, Wellons MF, Bagchi S, Chi BH, et al. Cardiometabolic risk factors among HIV patients on antiretroviral therapy. Lipids Health Dis 2013;12:50. 23575345 10.1186/1476-511X-12-50 PMC3641018

[10] Kaplan NM, Palmer BF, Terada LS, Gu Y, Flores SC. AIDS vasculopathy. Am J Med Sci 2000;320:379–87. 11149550 10.1097/00000441-200012000-00005

[11] Theetha Kariyanna P, Yager J, Salciccioli L, M. Lazar J, John Polman D, Priyan Chandrakumar H, et al. HIV-associated extracranial arterial aneurysms: a systemic review. Am J Med Case Rep 2020;8:128–33.32432158

[12] Heizer J, Petersen TC, Flemmer M. Multiple coronary aneurysms in a young adult with acquired immunodeficiency syndrome. Oxf Med Case Reports 2016;2016:109–12. 27168940 10.1093/omcr/omw036 PMC4860520

[13] Ayers J, Mandell R, Sanghvi K, Aboujaoude R, Hsi DH. Acute coronary thrombosis and multiple coronary aneurysms in a 22-year-old man with the human immunodeficiency virus. Tex Heart Inst J 2014;41:208–11. 24808786 10.14503/THIJ-13-3160 PMC4004470

[14] Sonia J, Khaldoun BJ, Sylvia M, Faouzi M, Habib G, Mohamed BF. Stenosis and aneurysm of coronary arteries in a patient with Behçet’s disease. Open Cardiovasc Med J 2008;2:118–20. 19430524 10.2174/1874192400802010118 PMC2627529

[15] Ouali S, Kacem SS, Ben Fradj F, Gribaa R, Naffeti E, Remedi F, et al. Takayasu arteritis with coronary aneurysms causing acute myocardial infarction in a young man. Tex Heart Inst J 2011;38:183–6. 21494533 PMC3066825

[16] Swaye PS, Fisher LD, Litwin PA, Vignola PA, Judkins MP, Kemp HG, et al. Aneurysmal coronary artery disease. Circulation 1983;67:134–8. 6847792 10.1161/01.cir.67.1.134

[17] Szalat A, Durst R, Cohen A, Lotan C. Use of polytetrafluoroethylene-covered stent for treatment of coronary artery aneurysm. Catheter Cardiovasc Interv 2005;66:203–8. 15977267 10.1002/ccd.20448

[18] Mahan VL, Balaguer JM, Pezzella AT, Salm TJV, Mady BJ. Successful coronary artery bypass surgery in a patient with AIDS. Ann Thorac Surg 2000;70:1698–9. 11093516 10.1016/s0003-4975(00)01692-1

[19] Boccara F, Cohen A, di Angelantonio E, Meuleman C, Ederhy S, Dufaitre G, et al. Coronary artery bypass graft in HIV-infected patients: a multicenter case control study. Curr HIV Res 2008;6:59–64. 18288976 10.2174/157016208783571900

