# Correction to: Coronary artery aneurysm, ectasia and stenosis in a 53-year-old man with HIV infection

**DOI:** 10.1093/jscr/rjac342

**Published:** 2022-07-12

This is a correction to: Abhishek Kashyap, Dmitry Abramov, Aditya Bharadwaj, Miriam Rabkin, David G. Rabkin, Coronary artery aneurysm, ectasia and stenosis in a 53-year-old man with HIV infection, *Journal of Surgical Case Reports*, Volume 2022, Issue 3, March 2022, rjac056, https://doi.org/10.1093/jscr/rjac056

In the originally published version of this manuscript, Abhishek Kashyap's first name was inadvertently misspelled as Abishek.

This error has been corrected.

